# Urachal xanthogranuloma: a rare but important case presenting as a urachal mass

**DOI:** 10.1186/s12894-023-01297-4

**Published:** 2023-08-03

**Authors:** Kunal Jain, Esha Jain, Richard DiLena, Rola Saleeb, Umesh Jain

**Affiliations:** 1 Section of Urology, Department of Surgery, University of Manitoba, AE101-820 Sherbrook St, University of Manitoba (Bannatyne Campus), Winnipeg, MB R3A 1R9 Canada; 2 Department of Family Medicine, Cooper Medical School of Rowan University, Camden, NJ Canada; 3 Division of Urology, Department of Surgery, University of Toronto, Toronto, ON Canada; 4 Department of Laboratory Medicine and Pathobiology, University of Toronto, Toronto, ON Canada

**Keywords:** Urachus, Xanthogranuloma, Urachal mass, Urology, Case report

## Abstract

**Background:**

A urachal mass is a relatively rare presentation to the urologists’ practice, often requiring radical surgical excision for a definitive diagnosis. Xanthogranulomatous inflammation of the urachus is an extremely rare entity with few cases reported worldwide, and to the best of our knowledge, no cases reported in the western world.

**Case presentation:**

In this case, a 55-year-old male patient presented with bothersome lower urinary tract symptoms and computed tomography findings demonstrating a urachal mass that was worrisome for urachal carcinoma. Following surgical intervention, histopathology revealed urachal xanthogranuloma. Post-operatively, the patient recovered well, and eventually, he had symptomatic and radiologic improvement.

**Conclusion:**

This case brings awareness to a rare presentation of a urachal mass—urachal xanthogranuloma. While operative intervention was both diagnostic and therapeutic, we highlight the challenge in differentiating between benign and malignant processes for urachal masses. Herein, we show the importance of including urachal xanthogranuloma in the differential diagnosis of a urachal mass to prevent further morbidity associated with the treatment of this disease.

## Background

Xanthogranulomatous disease is a form of chronic inflammation. Xanthogranulomatous inflammation of the urachus is an extremely rare entity with few cases reported worldwide, and to the best of our knowledge, no cases reported in the western world. Given the difficulty of distinguishing between carcinoma and xanthogranuloma on imaging or frozen section and the high rate of infection in granulomatous tissue, diagnosis is made by surgical excision and histopathologic examination [1, 2].

Herein, we present a 55-year-old male with a suspected urachal carcinoma. Following surgical excision, this mass was diagnosed as urachal xanthogranuloma.

## Case presentation

**Fig. 1:**
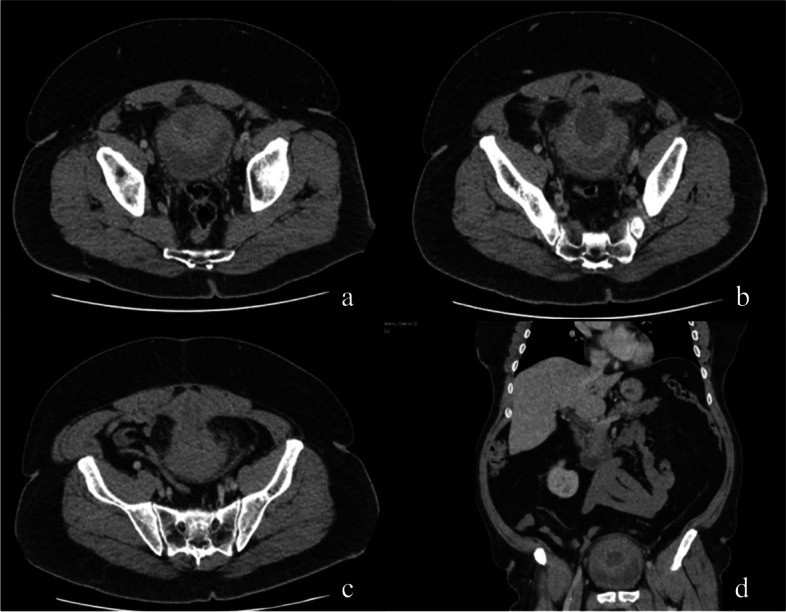
Pre-operative uninfused CT abdomen/pelvis showing a 10.3 cm solid and cystic mass abutting the superior bladder, extended up to the level of the umbilicus. **a**, **b**, **c** Cross-sectional, axial images showing the mass. **d** Coronal image showing the mass and omental nodularity

A 55-year-old Argentinian male was referred to a Canadian urologist’s practice in February 2021 with a three-month history of left lower abdominal pain, urinary frequency, nocturia, and urgency. The patient denied bothersome obstructive lower urinary tract symptoms, gross hematuria, dysuria, urinary incontinence, and B-symptoms including weight loss and change in appetite. His past medical history was significant for obesity and a laparoscopic cholecystectomy in 2017. He had no known drug allergies or medications. Physical examination demonstrated a patient with truncal obesity, weight of 125 kg, height of 178 cm, no abdominal masses, unremarkable umbilicus, unremarkable male genitalia, and a benign prostate. Screening hematologic, extended electrolytes, creatinine, blood urea nitrogen, glomerular filtration rate, and prostate-specific antigen levels were unremarkable. Urinalysis, urine culture, and urine cytology were negative. Abdominal ultrasound demonstrated an unremarkable post-void residual and prostate size but an abnormal 4.2 × 6.1 × 5.3 cm mass near the superior aspect of the bladder, prompting further investigation. Cystoscopy was unremarkable and did not reveal a bulge at the dome. Computed tomography (CT) abdomen/pelvis revealed a 10.3 cm solid and cystic mass abutting the superior bladder and extending up to the level of the umbilicus. There was associated mild soft tissue thickening, stranding, and nodularity on the anterior aspect of retro-umbilical omentum (Fig. 1a, b, c, d). The radiologist’s impression was a potential underlying malignancy, likely a urachal carcinoma, with suspected focal omental carcinomatosis.

**Fig. 2:**
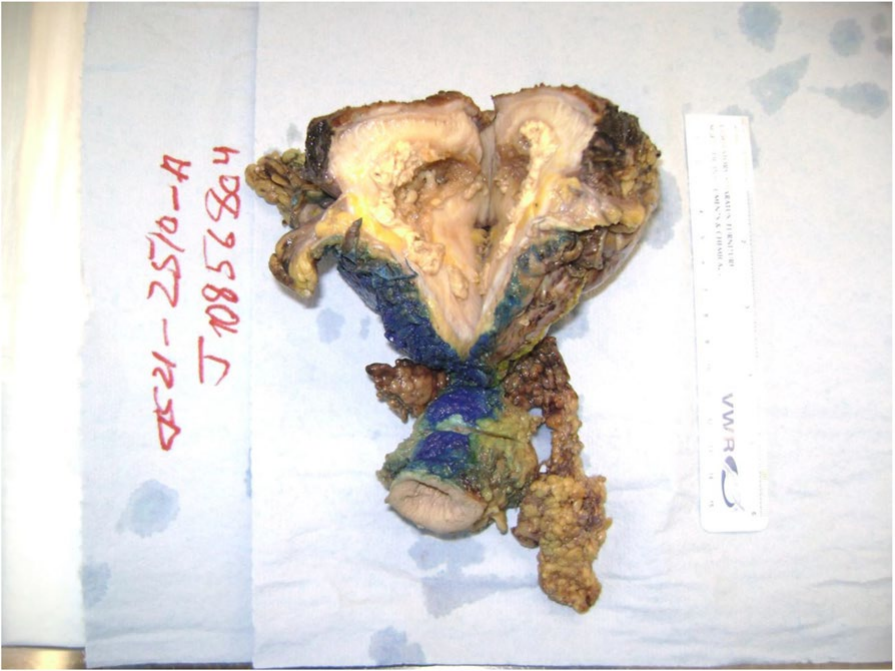
Gross pathology specimen of the 14.5 × 13 × 9.5 cm urachal mass abutting the bladder (top) to the umbilicus (bottom)

**Fig. 3:**
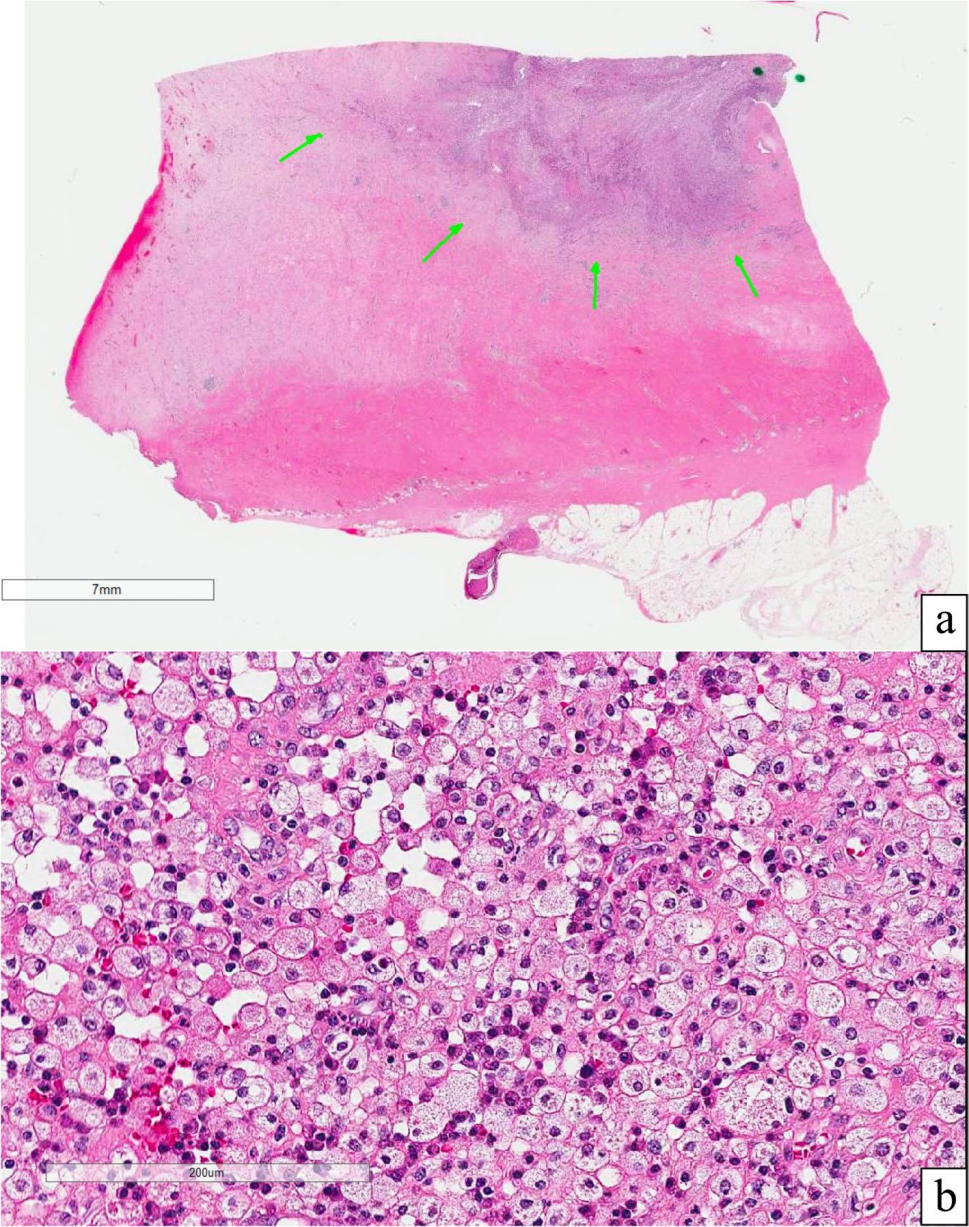
Histologic evaluation of the urachal mass. **a** Low power H&E stain with green arrows pointing toward a section of the xanthogranulomatous mass. **b** High power H&E stain of the xanthogranulomatous mass showing its characteristic foamy macrophages

In March 2021, the patient underwent an open surgical excision of a urachal mass, partial cystectomy, and bilateral extended pelvic lymph node dissection. Gross pathology revealed a 14.5 × 13 × 9.5 cm mass abutting the superior aspect of the bladder (Fig. 2). Microscopically, this mass was composed of mixed inflammatory cells with foamy macrophages (Fig. 3a, b). There was a negative Von Kossa stain for malakoplakia, a negative Ziehl–Neelsen-GMS stain for microorganisms, a positive CD68 immunostain for macrophages, and a negative pankeratin stain to confirm no dysplastic or malignant cells. Additionally, all nodes sampled revealed small, scattered foci of granulomatous inflammation (3/3 left external iliac, 8/8 left internal iliac, 6/6 right external iliac, and 9/9 right internal iliac) with no evidence of dysplasia or metaplasia. These findings were consistent with a xanthogranulomatous mass with nodal extension.

Post-operatively, the patient had an uncomplicated hospital course. His two-week post-operative CT cystogram did not reveal a bladder leak; thus, his foley was discontinued and the patient passed his trial of void. At his two-month follow-up visit, he had returned to his baseline health with resolution of his urinary symptoms. Follow-up imaging revealed the absence of the urachal mass and no further worrisome findings.

## Discussion and conclusions

The urachus is an embryological remnant of the allantois that normally obliterates during the 5th to 7th month of gestation to ultimately form the median umbilical ligament [3]. It is relatively uncommon to have defects during the obliteration process. Urachal disorders often present with symptoms in adulthood, with infection being the most common complication [4].

Xanthogranulomatous inflammation is pathologically characterized by the accumulation of lipid-laden foamy macrophages. While various sites may be affected, xanthogranulomatous pyelonephritis is the most common urologic presentation. Urachal xanthogranulomatous inflammation is exceedingly rare worldwide with no known reported cases in North America and few reported cases from Japan, Korea, and Spain [5]*.*

CT is used as an initial diagnostic modality to help diagnose urachal masses. CT findings of urachal xanthogranuloma often reveal a mass that is characterized as cystic, solid, or a solid-cystic lesion in the dome of the bladder [5, 6]. Differentiating between urachal xanthogranuloma and urachal carcinoma on CT is challenging, as seen in our case. There is not enough evidence to use magnetic resonance imaging to differentiate between the two pathologies [7]. Urachal carcinoma may consist of two parts, the supravesical and intravesical portions. The intravesical portion is seen as an unencapsulated protrusion from the bladder that is papillary or polypoid in nature whereas the supravesical portion is encapsulated [7]. The similarity on current imaging techniques between xanthogranulomatous inflammation and the supravesical portion of a urachal carcinoma necessitates early surgical intervention for definitive histopathology.

While no guidelines exist to support clinicians in managing urachal xanthogranulomas, the current consensus in managing suspected urachal carcinoma is en bloc surgical removal of the umbilicus, urachal ligament, and partial cystectomy with pelvic lymphadenectomy [8]. This intervention is necessary for accurate staging. Comparatively, management for urachal xanthogranuloma may not require lymphadenectomy, which could minimize associated morbidity. In our case, however, granulomatous disease was evident in the lymph nodes, although the utility of lymphadenectomy in this case is unknown. Additionally, urachal carcinoma carries a poor prognosis with a five-year overall survival of 50%, [9] whereas a review of published case reports of urachal xanthogranuloma demonstrates minimal long-term morbidity and mortality; thus, aggressive extended surgical intervention may not be necessary in urachal xanthogranuloma.

In conclusion, xanthogranulomatous inflammation of the urachus is a rare but important entity to include in the differential diagnosis of urachal masses. Our case study highlights the challenge in differentiating between urachal carcinoma and urachal xanthogranulomas. It is evident, not only from our case but also from the few other cases reported globally that this mass is easily misdiagnosed on imaging and requires definitive diagnosis with surgical excision and histopathology. Ultimately, surgery was both diagnostic and therapeutic; however, there is a need for earlier, more accurate diagnosis to minimize additional interventions and morbidity associated with aggressive surgical treatment. This case is especially important to urologists globally, as it will hopefully inform them about the diagnosis and management of urachal xanthogranulomas and spur advancements in differentiating it from malignant conditions.

## Data Availability

Data is accessible through the patient’s clinical chart, which requires confidential hospital-controlled access.

## Abbreviation

CT: Computed tomography

## References

[1] Park S, Ji YH, Cheon SH (2009). Urachal Xanthogranuloma: Laparoscopic Excision with Minimal Incision. Kor Jour of Uro.

[2] Tseng SF, Yang WC, Sung MT (2010). Urachal Xanthogranulomatous Inflammation. Urol Sci.

[3] Singh A, Kishan Prasad HL, Shetty KJ (2008). Urachal cyst with xanthogranulomatous cystis. Urology Annals.

[4] Nimmonrat A, Na-ChiangMai W, Muttarak M (2008). Urachal abnormalities: clinical and imaging features. Singapore Med J.

[5] Aiyappan SK, Vinchurkar KN, Shanmugan V (2020). Xanthogranulomatous Urachitis. Indian J Surg.

[6] Candamio M, Pombo F, Arnal F, et al. Clinical Image. Xanthogranulomatous Urachitis: CT Findings. J Comput Assist Tomogr. 1998;22(1):93–95. 10.1097/00004728-199801000-00018 9448770

[7] Lee SH, Kitchens HH, Kim BS (1990). Adenocarcinoma of the Urachus: CT Features. J Comput Assist Tomogr.

[8] Hamilou Z, North S, Canil C (2020). Management of urachal cancer: A consensus statement by the Canadian Urological Association and Genitourinary Medical Oncologists of Canada. Can Urol Assoc J.

[9] Pinthus JH, Haddad R, Trachtenberg J (2006). Population based survival data on urachal tumors. J Urol.

